# In Silico Modeling of Spirolides and Gymnodimines: Determination of *S* Configuration at Butenolide Ring Carbon C-4

**DOI:** 10.3390/toxins12110685

**Published:** 2020-10-29

**Authors:** Christian Zurhelle, Tilmann Harder, Urban Tillmann, Jan Tebben

**Affiliations:** 1 Department of Ecological Chemistry, Alfred Wegener Institute, Helmholtz Centre for Polar and Marine Research, Am Handelshafen 12, 27570 Bremerhaven, Germany; christian.zurhelle@awi.de (C.Z.); t.harder@uni-bremen.de (T.H.); urban.tillmann@awi.de (U.T.); 2 Faculty of Biology and Chemistry, University of Bremen, Leobener Straße 6, 28359 Bremen, Germany

**Keywords:** gymnodimines, spirolides, marine biotoxins, stereochemistry, shielding tensors, simulated NMR

## Abstract

Only few naturally occurring cyclic imines have been fully structurally elucidated or synthesized to date. The configuration at the C-4 carbon plays a pivotal role in the neurotoxicity of many of these metabolites, for example, gymnodomines (GYMs) and spirolides (SPXs). However, the stereochemistry at this position is not accessible by nuclear Overhauser effect—nuclear magnetic resonance spectroscopy (NOE-NMR) due to unconstrained rotation of the single carbon bond between C-4 and C-5. Consequently, the relative configuration of GYMs and SPXs at C-4 and its role in protein binding remains elusive. Here, we determined the stereochemical configuration at carbon C-4 in the butenolide ring of spirolide- and gymnodimine-phycotoxins by comparison of measured ^13^C NMR shifts with values obtained in silico using force field, semiempirical and density functional theory methods. This comparison demonstrated that modeled data support *S* configuration at C-4 for all studied SPXs and GYMs, suggesting a biosynthetically conserved relative configuration at carbon C-4 among these toxins.

## 1. Introduction

**Figure 1:**
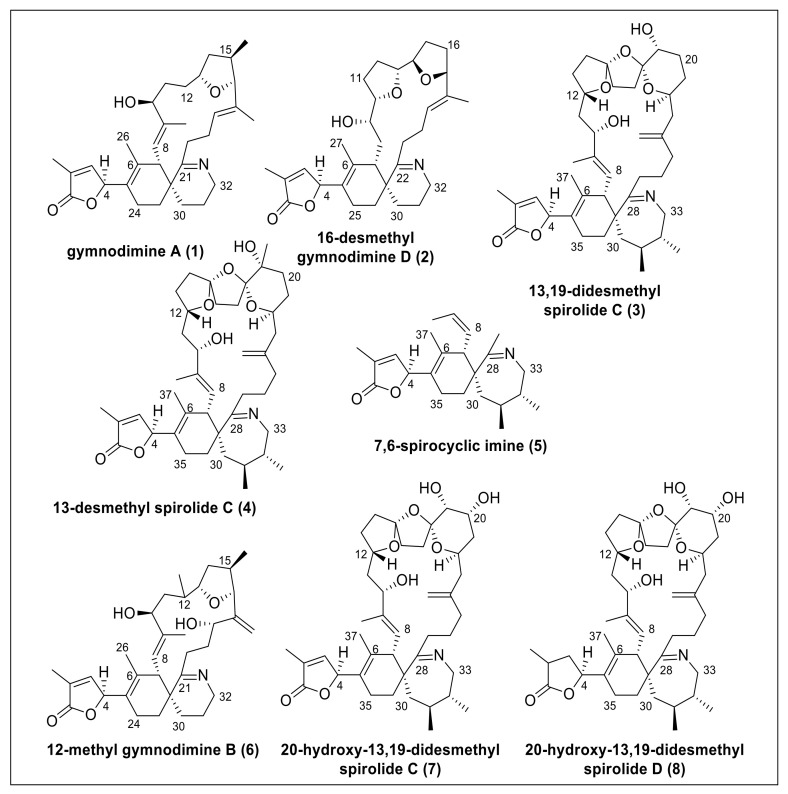
Structures of gymnodimines, spirolides, and 7,6-spirocyclic imine used in this study.

Macrocyclic imine (CI) phycotoxins are produced by several marine dinoflagellates and share the spiroimine structural motif [1]. The structural analogues of CI toxins pose no apparent oral toxicity to humans but administration by intraperitoneal injection results in lethal neurotoxic effects in mice [2]. CI toxins include several toxin classes (e.g., pinnatoxins, gymnodimines (GYMs), spirolides (SPXs), pteriatoxins) with striking structural diversity. These compounds are potent inhibitors of the nicotinic (nAChR) and muscarinic acetylcholine receptors [3]. The structural motifs most commonly linked to their bioactivity are the spiroimine and spiroketal rings [4]. Due to their prominent effect on nAChR, SPXs received attention as agents against neurodegenerative diseases [5,6,7]. SPXs and GYMs are highly similar CI analogues with several common structural features (Figure 1) because they are presumably encoded by the same genes [8]. Yet, only few naturally occurring CIs have been fully structurally elucidated or synthesized to date, giving rise to the notion if and how the stereochemistry of different CIs may influence receptor binding and bioactivity.

Most centers of chirality in GYMs and SPXs can be identified by nuclear magnetic resonance (NMR) measurements of the nuclear Overhauser effect (NOE). The configuration at the C-4 carbon, however, is not accessible by NOE-NMR due to unconstrained rotation of the single carbon bond between C-4 and C-5. Thus, the relative configuration of GYMs and SPXs at C-4 has remained elusive [9,10].

The stereochemistry at carbon C-4 plays a pivotal role in the toxicity of GYMs and SPXs because the configuration of the butenolide ring sterically interacts with the receptor protein [11]. The only structural analogues with confirmed relative stereochemistry at C-4 are: GYM A (**1**, determined by X-ray diffraction of the saturated imine derivate as *S* configuration [12] and synthesis [13]), 16-desmethyl GYM D (**2**, determined by circular dichroism (CD) spectroscopy as *S* configuration [14]), and 13,19-didesmethyl SPX C (**3**, determined by computational techniques as *R* configuration [9]). Additionally, X-ray diffraction analysis of 13-desmethyl SPX C (**4**) attached to nicotinic acetylcholine receptor [11] and NMR analysis of a synthesized partial substructure (7,6-spirocyclic imine, **5**) in comparison to the natural products of 13-desmethyl SPX C (**4**) and 13,19-didesmethyl SPX C (**3**) [10] suggested *S* configuration at C-4. Notably, Minamino et al. [10] argued that *R* configuration of 13,19-didesmethyl SPX C (**3**) proposed in silico by Ciminello et al. [8] was contradictory to their analysis of synthesized epimers of 7,6-spirocyclic imine (**5**). Together, these studies question if there is a conserved assignment of stereochemistry at C-4 among CI toxins.

Given this ambiguity, we addressed two objectives: (**1**) to develop a reliable in silico method to determine the relative configuration at C-4, and (**2**) to investigate if *S* configuration at carbon C-4 of the butenolide ring was a conserved feature of GYMs and SPXs. Specifically, we tested our approach on GYMs and SPXs with known configuration at carbon C-4, namely GYM A (**1**), 16-desmethyl GYM D (**2**), 13,19-didesmethyl SPX C (**3**), 13-desmethyl SPX C (**4**), 7,6-spirocyclic imine (**5**), and then applied our method on cyclic imines with unknown configuration at carbon C-4, i.e., 12-methyl GYM B (**6**), 20-hydroxy-13,19-didesmethyl SPX C (**7**), and 20-hydroxy-13,19-didesmethyl SPX D (**8**).

### Conceptual Idea

**Figure 2:**
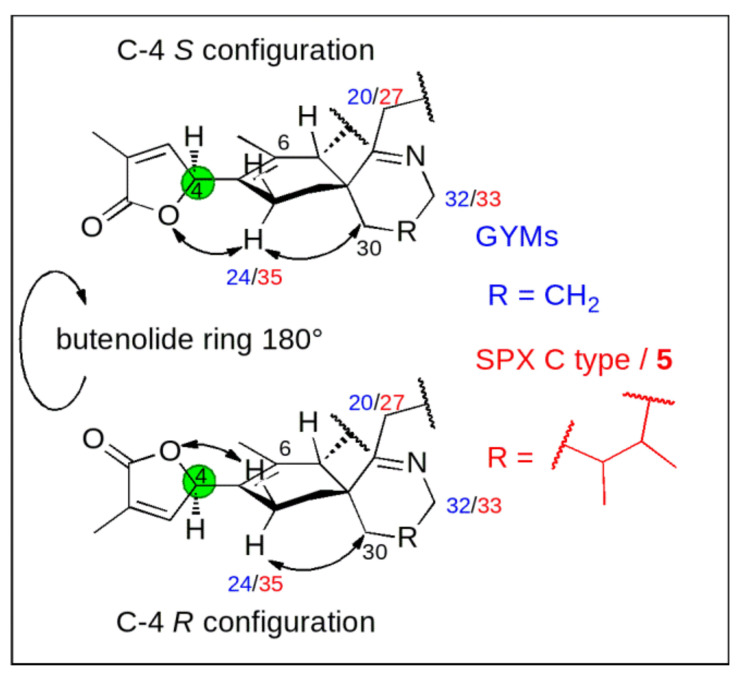
Effect of stereochemistry at carbon C-4 [green] on the chemical shift of the methylene carbon next to C-5 (C-24 in GYMs [blue] and C-35 in SPXs and 7,6-spirocyclic imine (**5**) [red]).

Conceptually, we measured ^13^C chemical shifts in SPXs and GYMs and compared these with calculated values obtained in silico using force field, semiempirical and density functional theory (DFT) methods both on the level of individual carbons as well as via the DP4+ approach [15]. The synthetic C-4 epimers of GYM A (**1**) and 7,6-spirocyclic imine (**5**) revealed a strong influence of C-4 stereochemistry on NMR shifts of neighboring nuclei, as such C-6 and C-24 in **1**, C-6 and C-35 in **5** showed the greatest difference of ^13^C chemical shifts [10,13]. This observation likely resulted due to the clear preference of the conformation with proton H-4 facing towards the methyl-group at cyclohexene ring B [8] (C-26 in **1**; C-37 in **5**, |α|_H-4,C-4,C-5,C-6_ = 40–60°, Appendix B Figure A2). Consequently, the spatial orientation of the oxygen between carbon C-1 and C-4 and the double bond (C-2, C-3) of the butenolide ring is determined by the conformational isomerism at C-4. The different chemical environment in combination with the spatial proximity of the imine ring and the methylene group at C-30 in **1** and **5** affect the chemical shift of C-24 in **1** and C-35 in **5** (Figure 2). The difference between the chemical shifts at C-6 is due to a similar effect; here the spatial proximity of C-6 and the stereochemistry at C-7 induce a different chemical environment at C-6 in both C-4 epimers. Therefore, we predicted C-4 stereochemistry by comparing experimental and simulated chemical shifts (∆δ = |δ_sim_ − δ_meas_|) at these distinct carbon positions in combination with DP4+ probability.

## 2. Results and Discussion

### 2.1. Simulation of Shielding Tensors

Representative geometries of each isomer are prerequisite for the simulation of shielding tensors [16,17,18]. Therefore, we used a diverse set of 200 possible geometries generated by a simulated annealing procedure with subsequent geometry optimization on the semiempirical level of theory (AM1). These geometries were ranked and binned in groups based on their dihedral angles by principle component analysis. Representative geometries with the lowest potential energy were chosen from each group, which had at least one geometry with a potential energy of maximal 10 kJ mol^−1^ higher than the conformer with the lowest potential energy. The representative geometries were subjected to geometry optimization and simulation of shielding tensors on the B3LYP level of theory including the conductor-like polarizable continuum model (CPCM) as the solvation model. The shielding tensors of each conformer were averaged based on the Boltzmann distribution and scaled by using the measured chemical shifts. The scaling factors were determined individually for each isomer. In addition to the comparison of the chemical shift at distinct positions, the DP4+ probability [15] was calculated.

The shielding tensors of 16-desmethyl GYM D were simulated at different levels of theory with and without prior optimization (Table 1). The objective was to achieve optimal accuracy at the lowest possible computational cost.

Shielding tensors obtained with B3LYP optimization showed the lowest root mean square errors (RMSEs) regardless of the application of the solvation model (Table 1). The MP2 theory improved the prediction for the nuclei C-6 and C-24 (GYM-A) in comparison to DFT, however, it did not improve the overall agreement between simulated and measured chemical shifts (Table 2). It is common that the application of MP2 does not improve the prediction of chemical shifts [19,20]. The difference in RMSE due to the application of CPCM as solvation model was minuscule. Given that the NMR data were obtained in different solvents [8,10,13,14,21,22,23,24], the application of a solvation model seemed advantageous nonetheless [25]. Therefore, DFT with the B3LYP functional and CPCM solvation model was applied for the simulation of shielding tensors in this study.

### 2.2. Individual Carbon Atoms

**Figure 3:**
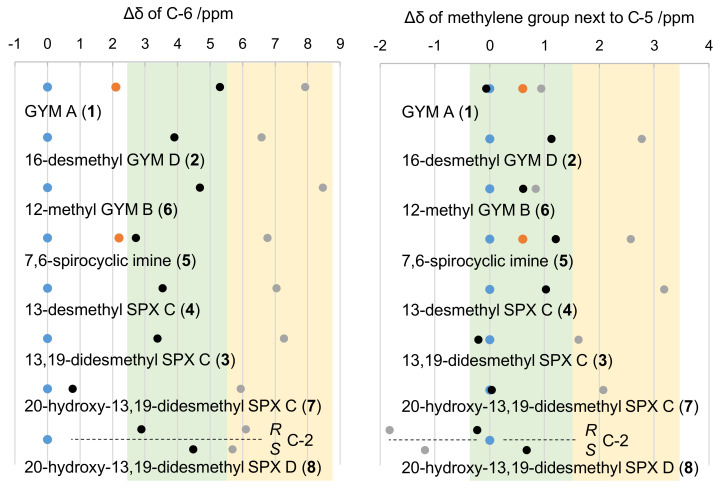
Chemical shifts of C-6 (left panel) and the methylene carbon adjacent to C-5 (right panel) for four SPXs, 7,6-spirocyclic imine, and three GYMs. Dots show ^13^C shifts relative to measured ^13^C shifts of the respective natural product (blue); simulated C-4 in *S* configuration (black), simulated C-4 in *R* configuration (grey); measured synthetic epimer in *R* configuration (orange). Relative chemical shift ranges of *S* and *R* epimers are shaded in green and yellow, respectively. The four C-2 isomers of 20-hydroxy-13,19-didesmethyl SPX D were modeled individually (separated by dotted line).

The simulation and comparison of individual NMR frequencies at carbon C-4 in *R* versus *S* configuration revealed a general downfield for ^13^C-shifts in *R* configuration, specifically for C-6 as well as the methylene carbon adjacent to C-5. This downfield shift was ~2.6 ppm for C-6 in **1**, ~4.4 ppm for **5**, ~1 ppm for the methylene carbon adjacent to C-5 in **1** (C-24), and ~1.4 ppm for the **5** (C-35). The simulated frequencies for C-24 in **1** were in good agreement with measured shifts of the natural product (Δ = 0.1 ppm for *S* and 0.3 ppm for *R*). For all other carbon atoms, however, the simulated shifts of both analogues showed higher simulated frequencies in comparison to measured data (Figure 3).

Although the predicted chemical shifts differed from measured values, their trends (up- or downfield shift) were predicted correctly (Figure 3). This allowed to assess the effect of carbon C-4 on spatially close nuclei of other SPXs and GYMs. Specifically, we addressed the hypothesis that a chemical shift towards higher frequencies, i.e., to lower magnetic field, at C-6 and the methylene carbon adjacent to C-5 is common for *R* epimers. This was clearly the case (Figure 3) as evidenced by simulations of the epimers of GYM A (**1**), 16-desmethyl GYM D (**2**), 13,19-didesmethyl SPX C (**3**), 13-desmethyl SPX C (**4**), 12-methyl GYM B (**6**), 20-hydroxy-13,19-didesmethyl SPX C (**7**), and 20-hydroxy-13,19-didesmethyl SPX D (**8**). Consequently, all GYMs and SPXs with known full relative stereochemistry, as well as three CI toxins with unidentified configuration at C-4 but high structural similarity to **4** or **1**, respectively, revealed a chemical shift towards higher frequencies as a conserved feature. Importantly, neither the imine ring size of GYMs and SPXs nor different ring systems in the side chain, e.g., in **1** and **4**, nor the hydration of the C-2, C-3 bond in **4** versus **8**, altered this characteristic trend of chemical shifts at C-6. Therefore, different structural motifs of the sidechain did not affect the chemical shift of C-6 at given stereochemistry of C-4. The same applied to the methylene carbon adjacent to C-5 among all CI toxins under investigation. Moreover, the predicted chemical shifts for the methylene group of the respective C-4 *S* epimers were either in agreement with or at lower field compared to measured data. The only exception to this trend was observed in predictions with **8**. This is likely due to the deshielding effect of the double bond between C-2 and C-3 on the carbon of the methylene group adjacent to C-5. The chirality of **7** resembled that of **4** with *R* configuration at C-20 based on NOE-NMR (Appendix C Figure A3). The high similarity of chemical shifts between **7** and **8** suggested the same stereochemistry of both compounds.

### 2.3. DP4+ Probability

DP4+ weighs the accuracy of predicted chemical shifts, resulting in a higher score for lower ∆δ values, based on Student’s *t*-distribution [15]. The DP4+ probability indicated *S* configuration at C-4 of all compounds under investigation (Table 3). 12-methyl GYM B showed the lowest probability (93.27%) and was therefore above the confidence threshold of 90% [15].

The additional chirality center at C-2 of 20-hydroxy-13,19-didesmethyl SPX D adds an additional degree of freedom to this compound. As for individual chemical shifts, all possible combinations of C-2 and C-4 were included in the calculations. The results suggested that 20-hydroxy-13,19-didesmethyl SPX D had *R* configuration at C-2 and S configurations at C-4 (99.99%). In conclusion, DP4+ probability assigned C-4 *S* configuration to all GYMs and SPXs considered in this study.

### 2.4. Re-Examination of C-4 in **3**

**Figure A1:**
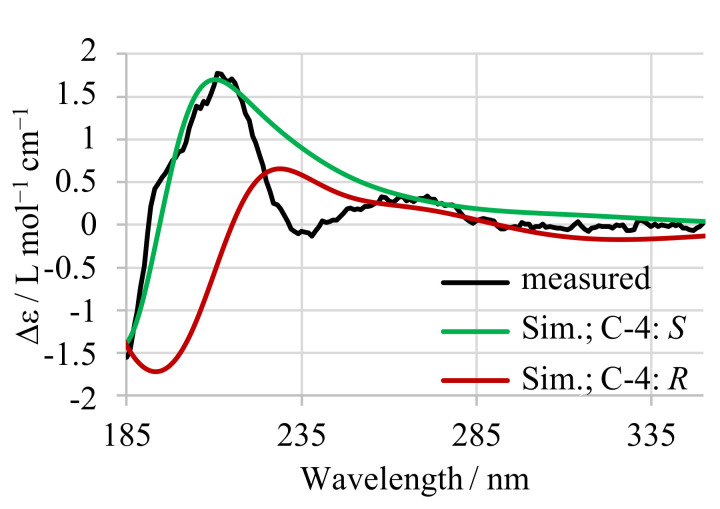
Measured CD-spectrum (black) of 13-desmethyl SPX C with simulated spectra for both configurations at C-4 (*S* in green, *R* in red).

**Figure A2:**
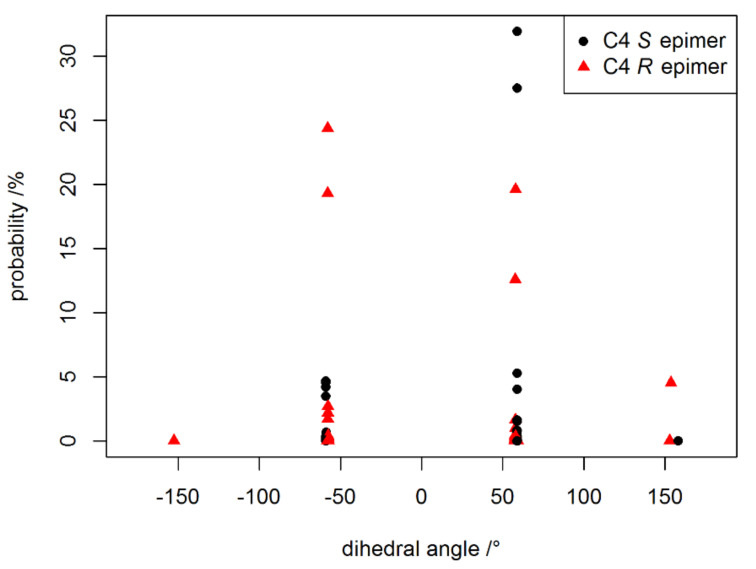
The probability of a geometry to occur according Boltzmann (292 K) versus dihedral angle α between H-4, C-4 and C-5,C-6.

**Figure A3:**
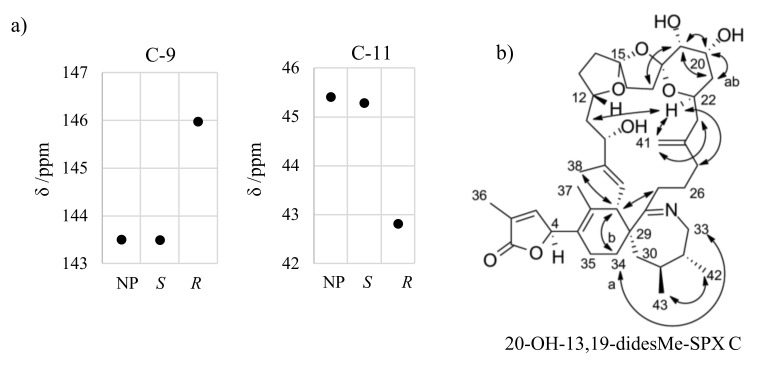
Simulated and measured chemical shift of C-9 and C11 for 20-hydroxy-13,19-didesmethyl SPX C (**a**) and important ROESY correlations for assignment of stereochemistry of 20-hydroxy-13,19-didesmethyl SPX C (**b**).

The comparison of individual chemical shifts and DP4+ probability suggested *S* configuration at C-4 as a conserved feature among all studied GYMs and SPXs. This result corroborated earlier studies showing *S* configuration at C-4 with other methodologies, such as X-ray diffraction (**1** [12]), CD spectroscopy (Appendix A **4** Figure A1, **2** [14]), and chemical synthesis (**5** [10]).

Yet, this conclusion disagreed with the stereochemistry reported for C-4 in **3** [8]. In their study, Ciminiello et al., assigned the configuration to C-4 by considering single conformers [8], concluding that among modeled conformers, the gauche conformer with *R* configuration at C-4 (α = −53°; Table 3) was most plausible, whereas the *S* conformer was incompatible with the spatial proximity of H-4 and H-35b.

We reexamined the data for **3** [8] according to Bifulco et al. [16] because the Boltzmann distribution (Table A1) suggested that **3** did not have dominant conformers and therefore required the averaging of multiple conformers. When applying this rule, the modeled average distances between H-4 and H-35b were either the same or shorter among C-4 *S* epimers than *R* epimers (Table A1), indicating *S* configuration at C-4. The simulation of NMR shifts in silico, the comparison of simulated spatial distances in comparison to NOE signals (both this study), and the interpretation of a partial structural motif of spirolides [10] suggested *S* configuration at C-4 for **3**.

## 3. Conclusions

The in silico assessment of the C-4 stereochemistry indicated *S* configuration for all GYMs and SPXs tested in this study. Our data confirmed previous results obtained by X-ray diffraction, synthesis, and CD spectroscopy (13-desmethyl SPX C (**4**), GYM A (**1**) and 16-desmethyl GYM D (**2**) [10,11,12,13,14]). Our data revised the C-4 stereochemistry of 13,19-didesmethyl SPX C (**3**) and annotated the C-4 stereochemistry for 12-methyl GYM B (**6**), 20-hydroxy-13,19-didesmethyl SPX C (**7**), and 20-hydroxy-13,19-didesmethyl SPX D (**8**). Our results highlight that *S* stereoisomerism at C-4 is likely a conserved feature of these toxins.

**Figure A4:**
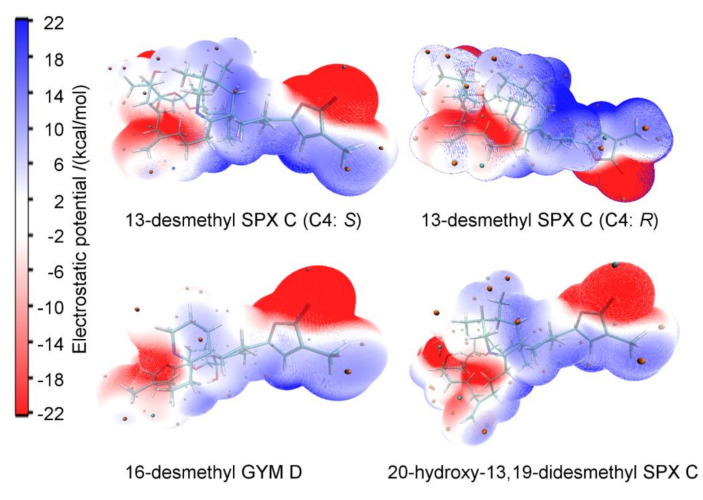
The electrostatic potential (ESP) surface of 13-desmethyl spirolide C for both C4 epimers (*S* left; *R* right), 16-desmethyl GYM D and 20-hydroxy-13,19-didesmethyl SPX C.

Since many marine-derived natural products are often isolated in minute amounts and possess complex and/or remote chiral centers within the molecules, the process of determining correct stereochemical assignments is often very challenging. The correct assignment of natural products is critical to many researchers since potent bioactive marine natural products often become synthetic targets, pharmaceutical leads, or molecular probes to characterize biological functions. These studies require accurate electron density models (Appendix D Figure A4: electrostatic potential surface of 13-desmethyl SPX C, 20-hydroxy-13,19-didesmethyl SPX C and 16-desmethyl GYM D) and receptor binding simulations to identify the molecules’ mode of action and to extrapolate their toxicity.

## 4. Materials and Methods

### 4.1. Parameter Setup

Simulations were either performed on a Lenovo ThinkPad workstation or on the Linux cluster Cray CS400 “Ollie” at the Alfred Wegener Institute’s computing center. The initial three- dimensional coordinates for all stereoisomers of compounds under investigation were obtained with the Avogadro software package [26]. The data were used for Visual Molecular Dynamics (VMD) [27] and Swissparam [28] to obtain the force field parameters for a simulated annealing procedure in the Nanoscale Molecular Dynamics program (NAMD).

### 4.2. Conformational Search

A simulated annealing procedure [29] by Nanoscale Molecular Dynamics (NAMD) [30] was applied to search for conformers according to the simulation parameters described in Ciminiello et al. [8]. The simulation was performed with a time step length of 1 fs. The distance-dependent dielectric constant was set to the value of methanol (ε = 33 × r). After an initial geometry optimization, a total of 200 simulated annealing cycles were performed under the following settings: Equilibration (10 ps) at 300 K, stepwise temperature increase (ΔT = 10 K) with 4 ps equilibration at each step, equilibration (20 ps) at 1000 K, stepwise temperature decrease (ΔT = 20 K) with 4 ps equilibration at each step, equilibration (2 ps) at 300 K.

The geometries obtained in each cycle of the simulated annealing were subjected to geometry optimization with force field and semiempirical on AM1 level of theory [31]. The semiempirical AM1 structure optimization and frequency analysis were performed with the General Atomic Molecular and Electronic Structure System (GAMESS) software package [32].

The AM1 conformers were ranked by conformational energy values and grouped into families according to dihedral angles through principal component analysis (PCA). For each group within a tolerance of 10 kJ mol^−1^ above the lowest conformational energy, a representative geometry was chosen. The chosen geometries were geometry optimized on the B3LYP/ def2-TZVPP/RIJCOSX/def2/J level of theory in ORCA [33]. The deuterated solvents were simulated with the conductor-like polarizable continuum model (CPCM) [34] and parameters of non-deuterated solvents.

### 4.3. Determination of Shielding Tensors and NMR Chemical Shifts

Representative geometries were subjected to shielding tensor simulation in ORCA [33]. The simulation utilized the gauge-independent atom orbital model (GIAO) [35] with CPCM on the B3LYP/ def2-TZVPP/RIJK/def2/JK and the B3LYP/6-31+G** level of theory for individual chemical shifts and the DP4+ mechanism, respectively [15,36,37] (see SI for single point energies, shielding tensors and geometries). The shielding tensors of conformers were averaged by Boltzmann distribution of their potential energy. The DP4+ probabilities were determined under application of an Excel sheet, provided by Grimblat et al., (sarotti-NMR.weebly.com) [15]. For individual chemical shifts, the averaged shielding tensors of each nuclei were correlated with the respective NMR shift and based on a linear regression translated into a simulated chemical shift. By comparison of simulated and measured NMR shifts, the root mean square error (RMSE) was determined according to Formula (1):

(1) $$\mathrm{RMSE}=\sqrt{\frac{1}{N}\overset{N}{\underset{i=1}{\sum }}{\left(\delta_{i,\mathrm{simulated}}-\delta_{i,\mathrm{measured}}\right)}^{2}}.$$

### 4.4. Circular Dichroism Spectroscopy

The representative geometries of both C-4 isomers of 13-desmethyl SPX C were subjected to simulation of rotatory strengths applying time dependent DFT at the B3LYP level of theory with def2-TZVPP/def2/J, RIJCOSX approximation, and the CPCM solvation model (water). The simulated CD spectrum of 13-desmethyl SPX C was obtained by applying Gaussian broadening to each transition as previously described by Li et al. [38] and adjusted manually to the height of the experimental data.

### 4.5. Electrostatic Potential Surface

The wavefunction of both C-4 epimers was obtained with ORCA (DFT, B3LYP/def2-TZVPP7def2-JK) and analyzed with Multiwfn [39].

### 4.6. Reference NMR Spectra

Based on the simulated shielding tensors, NMR chemical shifts were simulated by comparison with published data of the respective compounds: 20-hydroxy-13,19-didesmethyl SPX C [14] (Supplementary Materials Table S1), and 20-hydroxy-13,19-didesmethyl SPX D [14] (Table S1); 13-desmethyl SPX C [23] (Table S1); 13,19-didesmethyl SPX C [8] (Table S1); 7,6-spirocyclic imine [10] (Table S2), GYM A [21,22] (Table S2 “C-4: S; NP”); C-4 R epimer of GYM A [13] (Table S2 “C-4: R; syn”); 12-methyl GYM B [24] (Table S2), 16-desmethyl GYM D [14] (Table S2). The ROESY spectra of 20-hydroxy-13,19-didesmethyl SPX C in MeOD were recorded in 1.7 mm microtubes at 292 K with an AVANCE II 600 MHz NMR spectrometer (Bruker) and a CPTCI micro-cryoprobe.

## Figures and Tables

**Table 1:** Tested levels of theory for optimization and determination of shielding tensors for 16-desmethyl GYM D (C-4: *S*) compared via root mean square error (RMSE).

Level of Theory		^13^C	Level of Theory		^13^C
Optimization	Shielding Tensors	RMSE	Optimization	Shielding Tensors	RMSE
B3LYP,CPCM	B3LYP,CPCM	2.02	none	B3LYP,CPCM	3.09
B3LYP	B3LYP	2.00	none	TPSSh, CPCM	3.20
B3LYP	PBE0	2.40	none	B3LYP	2.86
B3LYP	RHF	3.89	none	RHF	4.88
RHF	RHF	4.10			

**Table 2:** Comparison of simulated and measured chemical shifts of C-4 epimers of **1** by root mean square error (RMSE) at different levels of theory for all carbons and C-6 and C-24 only.

	DFT		MP2	
	RMSE_all_	RMSE_C-6,C-24_	RMSE_all_	RMSE_C-6,C-24_
*S* epimer	2.909	3.751	3.478	1.957
*R* epimer	3.030	5.637	3.284	3.404

**Table 3:** DP4+ probability for all studied compounds for the different C-4 epimers. For 20-hydroxy-13,19-didesmethyl SPX D, all four possible combinations of C-2 and C-4 stereochemistry were considered.

C-4 Configuration	*S*		*R*	
GYM A **(1)**	99.49%		0.51%	
12-methyl GYM B (**6**)	93.27%		6,73%	
16-desmethyl GYM D (**2**)	100.00%		0.00%	
7,6-spirocyclic imine (**5**)	96.21%		3.79%	
13-desmethyl SPX C (**4**)	100.00%		0.00%	
13,19-didesmethyl SPX C (**3**)	99.99%		0.01%	
20-hydroxy-13,19-didesmethyl SPX C (**7**)	100.00%		0.00%	
**C-2 Configuration**	***S***	***R***	***S***	***R***
20-hydroxy-13,19-didesmethyl SPX D (**8**)	0.01%	99.99%	0.00%	0.00%

## Supplementary Materials

The following are available online at https://www.mdpi.com/2072-6651/12/11/685/s1, Table S1. Measured chemical shifts of SPXs, Table S2. Measured chemical shifts of GYMs, Table S3. Simulated chemical shifts of 20-hydroxy-13,19-didesmethyl SPX D and 20-hydroxy-13,19-didesmethyl SPX C, Table S4. Simulated chemical shifts of 13,19-didesmethyl SPX C, 13-desmethyl SPX C and 7,6-spirocyclic imine, Table S5. Simulated chemical shifts of GYM A, 16-desmethyl GYM D and GYM E, and the single geometries with single point energies and shielding tensors (xlsx).

## Appendix A. CD Spectroscopy of 13-desmethyl SPX C

The configuration of C4 in 13-desmethyl SPX C was independently tested by simulation and measurement of circular dichroism (CD) spectra, as previously reported for 16-desmethyl GYM D [14]. The measured spectrum (Figure A1, black) was compared to simulated spectra of both C4 epimers (both B3LYP optimized). The experimental CD spectra and simulated CD spectra (Figure A1, *S* epimer: green, *R* epimer: red) of 13-desmethyl SPX C confirmed the *S* configuration at C-4.

## Appendix B. Reanalysis of 13,19-didesmethyl SPX C

**Table A1:** Dihedral angle values (α) and related intramolecular distances measured in AM1-modeled conformers of C-4 *R*- and *S*-isomers of 13,19-didesmethyl SPX C according to Ciminiello et al. [8] with their Boltzmann distribution (this study). Averaged intramolecular distances of 13,19-didesmethyl SPX C were obtained from AM1-modeled conformers (this study).

Family/Compound	Dihedral Angle α	H-3–H-37/Å	H-4–H-37/Å	H-4–H-35b/Å	Probability/%
C4: *R*					
13,19-didesmethyl SPX C: *anti*	168°	3.3	3.8	2.5	0.0
13,19-didesmethyl SPX C: *gauche^−^*	−53°	2.5	2.1	3.3	50.0
13,19-didesmethyl SPX C: *gauche^+^*	43°	4.7	2.3	3.7	50.0
13,19-didesmethyl SPX C: averaged		3.7 ± 0.5	1.9 ± 0.4	3.5 ± 0.4	
C4: *S*					
13,19-didesmethyl SPX C: *anti*	−176°	3.4	3.7	2.2	0.0
13,19-didesmethyl SPX C: *gauche^+^*	39°	2.3	2.3	3.7	58.0
13,19-didesmethyl SPX C: *gauche^−^*	−41°	4.2	2.4	3.7	42.0
13,19-didesmethyl SPX C: averaged		2.6 ± 0.7	2.2 ± 0.3	3.3 ± 0.2	
measured NOE strength		weak	strong	very weak	

## Appendix C. Stereochemistry of 20-Hydroxy-13,19-didesmethyl SPX C

The preliminary stereochemistry assignments for 20-hydroxy-13,19-didesmethyl-SPX C reduced the number of possible stereoisomers (cf. Figure A3 for important NOEs). The NOE between H-7 and H-38 indicated *E* configuration at the double bond between C-8 and C-9. A linkage between the stereo centers C-7 and C-29 was indicated by a NOE between H-7 and H-27, implying either *S* configuration at C-7 and *R* configuration at C-29, or both configurations inverted. However, the configuration (C-7 *S* and C-29 *R*) was indicated by the NOEs of H-34b to H-7 and H-34a to H-33. The strong NOE between H-42 and H-43 indicated an equatorial position of both methyl groups. These methyl groups of the modeled geometries of 13-desmethyl SPX C have an equatorial orientation. Therefore, both C-31 and C-32 were assigned with *S* configuration in analogy to 13-desmethyl SPX C.

Proton H-22 showed NOEs to other protons in the macrocycle (e.g., H-25, H-11), whereas H-17 and H-12 did not reveal such NOEs. Therefore, H-22 is directed towards the macrocycle and H-12 and methylene groups of the ether ring in the middle (C-16 and C-17) are directed away from the macrocycle. H-19 faces away from the macrocycle as indicated by the NOE between H-17 and H-19. Further, the proton H-19 showed a NOE to H-21 and H-20 in addition to a small coupling constant (^3^J_HH_ near 0 Hz). In conclusion, the proton H-19 has axial orientation, while an equatorial orientation is assigned to H-20. The NOE between H-22 and both protons of at C-25 indicated equatorial orientation for H-22. Therefore, *S* configuration was assigned at C-12 and *R* configuration at C-15, C-18, C-19, C-20, and C-22. The configuration at C-4 and C-10 is to be elucidated by the integration of in silico methods. In analogy to C-4, we focused on neighboring carbon atoms with a spatial proximity to another center of stereochemistry, namely C-9 and C-12. The simulated chemical shifts of C-9 and C-12 for the C-10 S epimer were in good agreement with the measured chemical shifts (Δδ*_S_* = 0.1 ppm; Δδ*_R_* = 2.5 − 2.6 ppm). Therefore, we suggest *S* configuration at C-10 for 20-hydroxy-13,19-didesmethyl SPX C.

## Appendix D. Electrostatic Potential Surface of SPXs and GYMs

The electrostatic potential (ESP) surface depict the size, shape, charge density, and site of chemical reactivity of molecules [40]. The comparison of ESPs for different GYMs and SPXs supports the observation of similar mode of actions of the two compound groups. Further, the change of stereochemistry at the C4 carbon has a strong effect on the ESP, as shown for 13-desmethyl SPX C (Figure A4).
