# Supplementary material for: In Silico Modeling of Spirolides and Gymnodimines: Determination of S Configuration at Butenolide Ring Carbon C-4
Source: Toxins (Basel). 2020 Oct 29;12(11):685. doi: 10.3390/toxins12110685 (PMC7692061; doi:10.3390/toxins12110685)
Supplement: Supplementary file 1 [file toxins-12-00685-s001.zip › toxins-969808 - supplementary.docx]

Supplementary Materials: In Silico Modeling of Spirolides and Gymnodimines: Determination of *S* Configuration at Butenolide Ring Carbon C-4

Christian Zurhelle, Tilmann Harder, Urban Tillmann and Jan Tebben

Reference Spectra

**Table S1.** Measured chemical shifts (all in ppm) of the ^13^C nuclei for the used spirolides.

|  | **20-OH-13,19-didesmethyl SPX D [13]** | **20-OH-13,19-didesmethyl SPX C [14]** | **13,19-didesmethyl**  **SPX C [8]** | **13-desmethyl**  **SPX C [21]** |
| --- | --- | --- | --- | --- |
| **Solvent** | **MeOD** | **C_5_D_5_N** | **CD_3_OD** | **CD_3_OD** |
| **Atom no.** | **C-4: unknown** | **C-4: unknown** | **C-4: *R*** | **C-4: unknown** |
| 1 | 182.3 | 175.7 | 176.5 | 176.8 |
| 2 | 36.3 | 130 | 130.6 | 131 |
| 3 | 35.7 | 148.3 | 149.6 | 149.5 |
| 4 | 79 | 80.9 | 82.5 | 82 |
| 5 | 129.7 | 124.9 | 125.8 | 126.4 |
| 6 | 131 | 133.5 | 134.1 | 133.2 |
| 7 | 47.6 | 47.6 | 48.5 | 48.1 |
| 8 | 123.4 | 123.6 | 125.1 | 122.5 |
| 9 | 144.8 | 143.5 | 143.8 | 146 |
| 10 | 76.6 | 76.4 | 77.2 | 76.8 |
| 11 | 44.8 | 45.4 | 45.7 | 45.2 |
| 12 | 79.6 | 79.4 | 80 | 79.8 |
| 13 | 32 | 31.8 | 32.3 | 32.8 |
| 14 | 37.2 | 37.2 | 37.8 | 38.2 |
| 15 | 118.8 | 117 | 117.8 | 118.1 |
| 16 | 34.6 | 34.8 | 35.2 | 35.2 |
| 17 | 36.2 | 35.5 | 34.5 | 32.1 |
| 18 | 110.9 | 110.3 | 109.4 | 112.2 |
| 19 | 71.2 | 71.3 | 72 | 71.1 |
| 20 | 69.8 | 69.3 | 29.4 | 35.7 |
| 21 | 37.8 | 38.4 | 31 | 29.9 |
| 22 | 64.4 | 63.7 | 69.5 | 69.1 |
| 23 | 46.2 | 46.9 | 47 | 46.3 |
| 24 | 146 | 147.5 | 147.1 | 145.6 |
| 25 | 37.1 | 35.6 | 35.9 | 34.6 |
| 26 | 24 | 23 | 23.2 | 21.8 |
| 27 | 35.8 | 34.9 | 35.7 | 36 |
| 28 | 181 | 174.4 | 178 | 201.3 |
| 29 | 52.1 | 52.7 | 51.2 | 52.4 |
| 30 | 36.4 | 37.5 | 37.9 | 36.7 |
| 31 | 36.7 | 35.8 | 37 | 37.5 |
| 32 | 39.2 | 40.6 | 41.1 | 38.8 |
| 33 | 51.9 | 52.8 | 52.9 | 51.8 |
| 34 | 33.2 | 31.4 | 32 | 32.4 |
| 35 | 20.2 | 19.2 | 20.1 | 20.3 |
| 36 | 14.5 | 10.7 | 10.5 | 10.5 |
| 37 | 16.4 | 16.7 | 17.2 | 16.7 |
| 38 | 12.2 | 12.6 | 12.2 | 12.9 |
| 39 |  |  |  |  |
| 40 |  |  |  | 22.7 |
| 41 | 113.5 | 110.6 | 110.8 | 112.6 |
| 42 | 19.2 | 20.1 | 19.7 | 18.9 |
| 43 | 19.2 | 20 | 20.4 | 20.1 |

**Table S2.** Measured chemical shifts (all in ppm) of the ^13^C nuclei for 7,6-spirocyclic imine and the used gymnodines.

|  | **7,6-spirocyclic imine [10]** | | **GYM A [19,20]** | | **16-desmethyl GYM D [14]** | **12-methyl GYM B [22]** |
| --- | --- | --- | --- | --- | --- | --- |
| **Solvent** | **CD_3_OD** | | **CDCl_3_** | | **C_5_D_5_N** | **C_5_D_5_N** |
| **Atom no.** | **C-4: *S*** | **C-4: *R*** | **C-4: *S*; NP** | **C-4: *R*; syn** | **C-4: *S*** | **C-4: unknown** |
| 1 | 176.8 | 176.7 | 174.8 | 174.5 | 175.5 | 175.3 |
| 2 | 131.2 | 131.4 | 130.3 | 130.7 | 130.2 | 130.2 |
| 3 | 149.5 | 150.1 | 147.1 | 147.5 | 148.6 | 148.8 |
| 4 | 82 | 81.3 | 80.3 | 79.7 | 81.6 | 81.3 |
| 5 | 126.3 | 126.2 | 124.8 | 124.6 | 125.9 | 125.0 |
| 6 | 133.9 | 136.1 | 132.9 | 135 | 136.0 | 134.1 |
| 7 | 46.7 | 46.4 | 46.1 | 46.2 | 43.6 | 46.1 |
| 8 | 128 | 128.1 | 127 | 127.3 | 31.8 | 125.3 |
| 9 | 130.7 | 130.3 | 139.7 | 139.5 | 71.6 | 142.9 |
| 10 |  |  | 79.5 | 79.5 | 83.4 | 77.2 |
| 11 |  |  | 31.7 | 31.7 | 27 | 39.9 |
| 12 |  |  | 32.5 | 32.4 | 24.9 | 35.7 |
| 13 |  |  | 77.8 | 77.7 | 78.6 | 83.6 |
| 14 |  |  | 37.5 | 37.5 | 82.5 | 38.4 |
| 15 |  |  | 37.8 | 37.2 | 29.4 | 36.8 |
| 16 |  |  | 89.7 | 89.5 | 32.1 | 92.1 |
| 17 |  |  | 134.4 | 134.4 | 82.9 | 156.3 |
| 18 |  |  | 124.5 | 124.4 | 133.1 | 70.9 |
| 19 |  |  | 22 | 21.8 | 124.9 | 34.6 |
| 20 |  |  | 31 | 30.9 | 21.9 | 31.1 |
| 21 |  |  |  | 171.8 | 31.8 | 174.3 |
| 22 |  |  |  | 41 | 173.3 | 41.9 |
| 23 |  |  | 33.6 | 33.5 | 42.7 | 32.9 |
| 24 |  |  | 19.3 | 19.9 | 33.6 | 19.8 |
| 25 |  |  | 10.7 | 10.7 | 19.7 | 11.0 |
| 26 |  |  | 17 | 17.3 | 11 | 17.0 |
| 27 | 45.3 | 45.3 | 11.1 | 11.1 | 18.1 | 12.0 |
| 28 | 202.4 | 202.5 | 20.2 | 20.1 |  | 17.3 |
| 29 | 50.4 | 50.1 | 14.6 | 14.5 | 15.3 | 17.6 |
| 30 | 38.2 | 39.1 | 26.4 | 26.5 | 26 | 112.5 |
| 31 | 36.4 | 36.4 | 20.7 | 20.6 | 20.5 | 26.4 |
| 32 | 39 | 38.9 | 49.6 | 49.7 | 50.3 | 21.3 |
| 33 | 52.6 | 52.9 |  |  |  | 49.5 |
| 34 | 31 | 30.6 |  |  |  |  |
| 35 | 20.3 | 20.9 |  |  |  |  |
| 36 | 10.5 | 10.5 |  |  |  |  |
| 37 | 17.1 | 17.7 |  |  |  |  |
| 38 | 14.4 | 14.3 |  |  |  |  |

Simulated Chemical Shift

**Table S3.** Simulated chemical shifts (all in ppm) of the ^13^C nuclei for 20-hydroxy-13,19-didesmethyl SPX D (all four combinations of possible configuration at C-2 and C-4) and 20-hydroxy-13,19-didesmethyl SPX C (both C-4 epimers with *S* configuration at C-10 and the C-4: *S*, C-10: *R* isomer).

|  | **20-OH-13,19-didesmethyl SPX D** | | | | **20-OH-13,19-didesmethyl SPX C** | | |
| --- | --- | --- | --- | --- | --- | --- | --- |
| **Atom no.** | **C-4: *S*,**  **C-2: *S*** | **C-4: *S*,**  **C-2: *R*** | **C-4: *R*,**  **C-2: *S*** | **C-4: *R*,**  **C-2: *R*** | **C-4: *S*,**  **C-10: *S*** | **C-4: *R*,**  **C-10: *S*** | **C-4: *S*,**  **C-10: *R*** |
| 1 | 174.3 | 175.2 | 174.9 | 174.1 | 168.6 | 167.9 | 169.1 |
| 2 | 37.2 | 37.0 | 36.1 | 37.5 | 132.6 | 133.0 | 133.1 |
| 3 | 36.5 | 34.1 | 34.9 | 35.4 | 143.8 | 145.5 | 144.4 |
| 4 | 77.8 | 77.9 | 74.6 | 76.0 | 79.8 | 78.5 | 80.3 |
| 5 | 131.8 | 132.0 | 132.4 | 130.3 | 128.5 | 127.4 | 129.5 |
| 6 | 135.5 | 133.9 | 136.7 | 137.1 | 134.3 | 139.4 | 134.8 |
| 7 | 50.5 | 48.9 | 49.3 | 49.6 | 49.0 | 51.3 | 50.7 |
| 8 | 122.5 | 127.0 | 126.8 | 127.4 | 125.7 | 120.9 | 123.1 |
| 9 | 145.5 | 143.6 | 143.3 | 143.3 | 143.5 | 144.3 | 146.0 |
| 10 | 77.1 | 79.7 | 79.8 | 79.6 | 79.0 | 76.4 | 73.3 |
| 11 | 41.0 | 46.4 | 46.2 | 46.2 | 45.3 | 41.0 | 42.8 |
| 12 | 83.2 | 79.8 | 79.8 | 79.7 | 79.2 | 82.4 | 77.8 |
| 13 | 32.6 | 30.5 | 30.7 | 30.6 | 30.3 | 32.6 | 31.9 |
| 14 | 37.6 | 37.0 | 37.3 | 37.3 | 37.0 | 37.6 | 38.9 |
| 15 | 121.2 | 119.3 | 119.2 | 119.1 | 118.3 | 119.7 | 120.7 |
| 16 | 33.8 | 35.7 | 35.5 | 35.6 | 35.5 | 33.7 | 33.3 |
| 17 | 33.3 | 36.5 | 36.4 | 36.5 | 36.6 | 33.4 | 32.8 |
| 18 | 114.1 | 114.3 | 114.2 | 114.2 | 113.4 | 112.8 | 111.9 |
| 19 | 70.2 | 72.3 | 72.2 | 72.2 | 72.0 | 69.5 | 69.4 |
| 20 | 71.2 | 71.3 | 71.4 | 71.4 | 70.8 | 70.7 | 71.1 |
| 21 | 36.9 | 37.2 | 37.2 | 37.3 | 37.2 | 36.8 | 37.2 |
| 22 | 64.1 | 64.6 | 64.6 | 64.7 | 64.4 | 63.7 | 63.0 |
| 23 | 48.0 | 49.3 | 49.0 | 49.0 | 49.2 | 47.8 | 48.4 |
| 24 | 152.8 | 151.3 | 150.8 | 151.0 | 150.0 | 150.7 | 150.6 |
| 25 | 33.8 | 35.9 | 36.2 | 36.1 | 36.6 | 33.6 | 35.2 |
| 26 | 21.8 | 22.2 | 22.0 | 23.3 | 24.3 | 21.8 | 21.9 |
| 27 | 34.6 | 35.7 | 35.9 | 35.8 | 36.1 | 34.4 | 35.2 |
| 28 | 175.4 | 174.4 | 174.1 | 174.5 | 172.9 | 173.6 | 173.4 |
| 29 | 52.3 | 52.3 | 52.2 | 52.2 | 51.9 | 51.8 | 52.3 |
| 30 | 37.3 | 36.7 | 37.3 | 37.3 | 36.6 | 37.5 | 37.3 |
| 31 | 36.2 | 36.0 | 35.9 | 35.8 | 35.8 | 35.9 | 36.1 |
| 32 | 42.4 | 42.3 | 42.6 | 42.5 | 41.9 | 42.7 | 42.5 |
| 33 | 53.4 | 52.8 | 52.9 | 52.8 | 52.4 | 53.0 | 53.2 |
| 34 | 32.7 | 32.3 | 31.8 | 31.9 | 31.9 | 32.5 | 32.5 |
| 35 | 20.9 | 20.0 | 19.0 | 18.4 | 19.2 | 21.3 | 20.0 |
| 36 | 13.5 | 15.0 | 14.9 | 13.3 | 10.1 | 11.1 | 10.7 |
| 37 | 16.5 | 15.6 | 16.3 | 16.3 | 15.8 | 17.2 | 16.6 |
| 38 | 14.5 | 8.7 | 9.2 | 8.9 | 9.2 | 15.3 | 16.5 |
| 39 |  |  |  |  |  |  |  |
| 40 |  |  |  |  |  |  |  |
| 41 | 106.4 | 107.1 | 107.0 | 107.8 | 107.6 | 105.3 | 106.7 |
| 42 | 18.2 | 17.4 | 17.9 | 17.2 | 17.3 | 18.3 | 18.3 |
| 43 | 18.7 | 18.2 | 18.7 | 18.1 | 18.1 | 19.1 | 18.8 |

**Table S4.** Simulated chemical shifts (all in ppm) of the ^13^C nuclei for 13,19-didesmethyl SPX C, 13-desmethyl SPX C and 7,6-spirocyclic imine (both C-4 epimers for each compound).

|  | **13,19-didesmethyl SPX C** | | **13-desmethyl SPX C** | | **7,6-spirocyclic imine** | |
| --- | --- | --- | --- | --- | --- | --- |
| **Atom no.** | **C-4: *S*** | **C-4: *R*** | **C-4: *S*** | **C-4: *R*** | **C-4: *S*** | **C-4: *R*** |
| 1 | 172.9 | 172.1 | 172.2 | 171.6 | 173.2 | 172.6 |
| 2 | 129.4 | 129.7 | 129.7 | 130.3 | 130.7 | 131.1 |
| 3 | 150.1 | 151.0 | 150.0 | 150.4 | 150.2 | 151.3 |
| 4 | 83.0 | 81.2 | 82.5 | 81.0 | 83.9 | 81.7 |
| 5 | 124.6 | 124.1 | 125.3 | 124.5 | 126.2 | 125.9 |
| 6 | 137.5 | 141.4 | 136.7 | 140.2 | 135.9 | 140.0 |
| 7 | 49.5 | 49.7 | 51.1 | 51.0 | 49.2 | 47.4 |
| 8 | 125.1 | 124.8 | 117.8 | 117.1 | 126.6 | 125.6 |
| 9 | 142.7 | 142.2 | 146.3 | 146.0 | 131.1 | 130.1 |
| 10 | 79.3 | 79.1 | 76.6 | 76.3 |  |  |
| 11 | 44.9 | 44.9 | 40.0 | 40.1 |  |  |
| 12 | 80.3 | 80.2 | 82.9 | 82.9 |  |  |
| 13 | 30.9 | 31.0 | 33.5 | 33.3 |  |  |
| 14 | 37.4 | 37.3 | 37.6 | 37.6 |  |  |
| 15 | 118.1 | 117.7 | 119.2 | 119.0 |  |  |
| 16 | 35.9 | 36.0 | 34.6 | 34.7 |  |  |
| 17 | 37.2 | 37.2 | 32.5 | 32.8 |  |  |
| 18 | 110.5 | 110.2 | 112.3 | 112.2 |  |  |
| 19 | 73.1 | 73.1 | 71.8 | 71.6 |  |  |
| 20 | 29.6 | 29.6 | 36.5 | 36.5 |  |  |
| 21 | 29.8 | 29.9 | 29.0 | 28.9 |  |  |
| 22 | 69.6 | 69.6 | 68.6 | 68.6 |  |  |
| 23 | 48.9 | 48.6 | 47.0 | 46.6 |  |  |
| 24 | 151.7 | 151.3 | 149.8 | 149.6 |  |  |
| 25 | 36.8 | 36.5 | 33.1 | 32.9 |  |  |
| 26 | 24.8 | 24.0 | 20.5 | 20.5 |  |  |
| 27 | 36.0 | 35.8 | 36.4 | 36.2 | 27.2 | 28.6 |
| 28 | 176.3 | 175.6 | 204.1 | 203.7 | 202.7 | 203.4 |
| 29 | 52.9 | 52.6 | 54.8 | 55.0 | 55.7 | 53.9 |
| 30 | 37.1 | 36.9 | 36.5 | 36.4 | 37.1 | 40.3 |
| 31 | 36.6 | 36.5 | 37.6 | 37.7 | 37.9 | 38.4 |
| 32 | 42.0 | 42.1 | 40.2 | 40.0 | 41.2 | 40.4 |
| 33 | 53.1 | 53.2 | 51.8 | 51.7 | 52.9 | 54.1 |
| 34 | 32.2 | 32.5 | 34.9 | 34.9 | 34.4 | 33.3 |
| 35 | 19.9 | 21.7 | 21.3 | 23.5 | 21.5 | 22.9 |
| 36 | 10.2 | 10.2 | 11.1 | 11.2 | 12.2 | 11.9 |
| 37 | 16.6 | 17.4 | 17.3 | 17.9 | 18.4 | 18.9 |
| 38 | 10.0 | 10.0 | 16.2 | 15.9 | 15.4 | 15.7 |
| 39 |  |  |  |  |  |  |
| 40 |  |  | 19.4 | 19.2 |  |  |
| 41 | 105.8 | 105.2 | 105.1 | 104.9 |  |  |
| 42 | 17.5 | 17.3 | 17.6 | 17.4 | 18.2 | 17.9 |
| 43 | 18.5 | 18.4 | 20.0 | 20.0 | 20.3 | 20.1 |

**Table S5.** Simulated chemical shifts (all in ppm) of the ^13^C nuclei for GYM A (with DFT and MP2, both C-4 epimers), 16-desmethyl GYM D (both C-4 epimers) and GYM E (both C-19 epimers with C-4: *S*).

|  | **GYM A** | | | | **16-desmethyl GYM D** | | **12-methyl GYM B** | |
| --- | --- | --- | --- | --- | --- | --- | --- | --- |
| **Atom no.** | **C-4: *S***  **DFT** | **C-4: *R***  **DFT** | **C-4: *S***  **MP2** | **C-4: *R***  **MP2** | **C-4: *S*** | **C-4: *R*** | **C-4: *S*** | **C-4: *R*** |
| 1 | 172.1 | 171.6 | 175.8 | 175.5 | 172.3 | 171.6 | 172.8 | 172.0 |
| 2 | 128.9 | 129.1 | 130.4 | 130.7 | 129.0 | 129.7 | 130.0 | 130.4 |
| 3 | 149.0 | 149.9 | 145.6 | 146.3 | 149.8 | 148.2 | 149.4 | 150.5 |
| 4 | 82.6 | 81.0 | 85.4 | 83.9 | 82.5 | 80.8 | 83.1 | 80.8 |
| 5 | 123.3 | 123.1 | 124.3 | 124.3 | 123.5 | 126.2 | 124.6 | 123.9 |
| 6 | 138.2 | 140.8 | 135.1 | 137.6 | 139.9 | 142.6 | 138.8 | 142.6 |
| 7 | 51.0 | 51.0 | 51.5 | 51.3 | 43.2 | 43.0 | 50.1 | 50.4 |
| 8 | 118.9 | 119.1 | 117.2 | 117.6 | 30.2 | 25.2 | 120.6 | 120.7 |
| 9 | 142.9 | 142.2 | 142.0 | 141.3 | 72.4 | 73.1 | 143.6 | 142.7 |
| 10 | 77.2 | 77.0 | 79.8 | 79.5 | 81.4 | 80.8 | 74.2 | 74.4 |
| 11 | 32.6 | 32.5 | 32.6 | 32.5 | 25.7 | 26.5 | 39.0 | 39.2 |
| 12 | 33.6 | 33.6 | 34.1 | 33.9 | 29.7 | 29.2 | 37.2 | 37.0 |
| 13 | 79.5 | 79.4 | 83.1 | 82.9 | 79.9 | 79.4 | 83.8 | 83.7 |
| 14 | 36.9 | 36.7 | 37.7 | 37.6 | 79.5 | 79.2 | 39.3 | 39.4 |
| 15 | 40.4 | 40.1 | 39.9 | 39.6 | 27.7 | 27.4 | 38.9 | 38.6 |
| 16 | 90.0 | 89.9 | 94.2 | 94.0 | 32.1 | 31.7 | 92.1 | 92.0 |
| 17 | 137.0 | 136.6 | 136.9 | 136.7 | 82.6 | 82.0 | 156.1 | 155.9 |
| 18 | 121.7 | 121.3 | 120.5 | 120.0 | 136.4 | 136.4 | 71.4 | 70.8 |
| 19 | 21.5 | 21.5 | 20.1 | 20.0 | 123.6 | 122.6 | 34.1 | 33.6 |
| 20 | 31.0 | 31.1 | 30.6 | 30.5 | 23.2 | 23.0 | 27.9 | 27.9 |
| 21 | 172.7 | 172.3 | 168.8 | 168.5 | 32.7 | 32.0 | 175.1 | 174.4 |
| 22 | 47.9 | 47.4 | 47.6 | 46.6 | 173.3 | 172.0 | 47.1 | 46.7 |
| 23 | 31.0 | 31.4 | 31.1 | 32.2 | 46.8 | 48.4 | 30.9 | 30.8 |
| 24 | 19.2 | 20.2 | 17.6 | 18.4 | 32.4 | 36.4 | 19.2 | 20.6 |
| 25 | 9.8 | 9.9 | 9.6 | 9.5 | 20.8 | 22.5 | 10.3 | 10.4 |
| 26 | 16.7 | 17.1 | 16.2 | 16.5 | 10.9 | 10.5 | 16.7 | 17.5 |
| 27 | 18.8 | 18.6 | 18.4 | 18.0 | 17.4 | 14.3 | 18.3 | 18.4 |
| 28 | 18.4 | 18.3 | 18.5 | 18.3 |  |  | 14.4 | 14.4 |
| 29 | 12.3 | 12.3 | 12.0 | 11.8 | 13.9 | 13.6 | 17.3 | 17.4 |
| 30 | 23.0 | 23.5 | 22.5 | 23.7 | 23.1 | 29.9 | 110.4 | 110.3 |
| 31 | 18.7 | 18.8 | 17.3 | 17.5 | 20.5 | 19.7 | 23.2 | 23.0 |
| 32 | 50.4 | 50.2 | 51.0 | 50.7 | 51.4 | 50.1 | 18.9 | 18.8 |
| 33 |  |  |  |  |  |  | 50.4 | 50.3 |
